# Testing for the “Blues”: Using the Modified Emotional Stroop Task to Assess the Emotional Response of Gorillas

**DOI:** 10.3390/ani12091188

**Published:** 2022-05-06

**Authors:** Jennifer Vonk, Molly McGuire, Jessica Leete

**Affiliations:** 1 Department of Psychology, Oakland University, Rochester, MI 48309, USA; jessie4ann@gmail.com; 2 Zoo Miami, Miami, FL 33177, USA; molly.mcguire@miamidade.gov

**Keywords:** gorilla, emotional Stroop, primate, valence, attentional shift, selective attention

## Abstract

**Simple Summary:**

We presented three adult male zoo-housed gorillas (*Gorilla gorilla gorilla*) with a modified emotional Stroop task on a computer touchscreen as a way to assess gorillas’ attentional shift for positively versus negatively valenced items. The gorillas learned to respond to a blue border and to withhold responding to a yellow border. Images of items assumed to have a positive or negative valence for the gorillas were placed within the borders. As predicted, gorillas touched the reinforced blue border more slowly when ‘negative’ images appeared within the blue border, compared to when ‘positive’ images appeared within the yellow border. However, gorillas’ accuracy did not vary as a function of which images appeared within the blue border, possibly because of the high levels of performance on all trials. These results validate the procedure to some degree for assessing the emotional valence of familiar stimuli.

**Abstract:**

We adapted the emotional Stroop task developed for primates to test whether gorillas would show response slowing for images of ‘negative’ compared to images of ‘positive’ items placed within previously reinforced borders. Three zoo-housed male gorillas participated in six phases of an emotional Stroop paradigm. In Phase One, they learned to select blue borders over yellow borders in a forced choice task presented on the touchscreen. In Phase Two, neutral yellow or blue two-dimensional shapes were placed within the borders. On congruent trials, blue images were presented within both blue and yellow borders. On incongruent trials, yellow images were placed within both blue and yellow borders. We continued to use these trials as control trials in subsequent phases. We predicted that response latencies would be slower and accuracy would be lower on incongruent trials. Although the gorillas responded more quickly to incongruent trials, in contrast to predictions, they were more accurate on congruent trials, consistent with predictions. Therefore, we proceeded with Phase Three in which photographs of images assumed to have positive and negative valences for the gorillas were placed within the borders. On test trials, the same positive or negative image was placed within both borders. In Phase Four, a positive image was paired with a negative image on each trial and the positive image appeared in either the blue (congruent trials) or yellow border (incongruent trials). Phases Five and Six replicated Phases Three and Four with images of novel positive and negative items. The gorillas responded more quickly on congruent trials compared to incongruent trials on test trials but not on control trials throughout Phases 3–6. These findings provide some validation for the emotional Stroop task to test attentional shift with emotionally valenced items.

## 1. Introduction

Empirical efforts to assess the emotional well-being of animals in human care have benefitted from recent methodological and technological advances to assess emotion states in nonverbal organisms. Because animals are nonverbal, researchers must rely on behavioral indicators to infer emotion states and the extent to which different emotion states are evoked by objects and contexts that animals may be exposed to. The current study aimed to assess the responses of three Western lowland gorillas (*Gorilla gorilla gorilla*) to various familiar objects using a modified pictorial emotional Stroop paradigm [1,2]. This paradigm tests how emotionally valent visual stimuli may disrupt attention through response slowing and reduced accuracy in selecting previously rewarded stimuli. Thus, the emotional Stroop task can be used to assess how potentially emotionally valent stimuli disrupt cognitive performance, providing a measure of the emotional salience of the stimuli. Therefore, in addition to providing an indication of an animal’s emotional reactivity, the emotional Stroop task can be used to assess whether objects elicit positive or negative responses as a form of preference test. Emotion states are linked to preferences in that positive affect should be evoked by the presence of preferred objects and the absence of non-preferred objects, whereas the reverse should be true for negative affect.

Animal caretakers often make assumptions about animals’ preferences that may not be valid [3,4,5] or stable, so it is important to find more objective methods for assessing preferences for a wide array of objects and experiences [6]. The method of presenting images on a touchscreen to assess preferences in primates has the advantage that preferences for things that cannot easily be displayed physically, such as different habitats and potential mates, can be assessed in the absence of any risks [7]. Whereas preferences for foods [6,7,8,9,10,11,12,13] and sounds [14,15,16] have been studied in gorillas, there have been few attempts to examine their emotional response to a wider array of familiar items. Previous attempts to train the same gorillas tested here to report their degree of preference for items using a nonverbal Likert scale [7] proved challenging, probably because success required the gorillas to perform a conditional discrimination based on the abstract construct of preference. These challenges led to the current approach because the emotional Stroop task required the gorillas to perform only a simple color discrimination, and previous studies had demonstrated its potential with other apes [17,18,19].

The Stroop task has a long history of use in human cognitive research, e.g., [20,21,22,23]. In the original version of the task, participants were slower to read color words printed in a different color versus a matching color [24]. In the emotional Stroop version of the task, naming or classifying the color of stimuli presented visually is affected by the presentation of emotionally valenced distractor stimuli (i.e., negative words or images) such that responses are slower [25] and less accurate [26]. For example, humans are slower to name colors of negative stimuli [20,27]. These effects are attributed to selective attention being drawn to emotionally valent stimuli or “emotional intrusion” [27].

The first study to use a modified emotional Stroop task in nonhuman primates [17], trained chimpanzees (*Pan troglodytes*), to select open blue borders when paired with open yellow borders (or vice versa) on a touchscreen. Once the chimpanzees reached a criterion level of responding, the researchers introduced a “proof of concept” phase whereby objects colored the same shade of blue or yellow were placed inside the borders. On congruent trials, yellow objects appeared within the yellow border and blue objects appeared within the blue border. On incongruent trials, blue objects appeared within yellow borders and yellow objects appeared within blue borders. The researchers predicted that responses would slow and accuracy would decline on incongruent compared to congruent trials because the images within the borders did not match the color of the border. This pattern of responding would verify that images within the borders led to shifts in selective attention. In the final test, the chimpanzees were presented with photographs of humans assumed to evoke different emotional responses (i.e., familiar caretaker, stranger, veterinarian) within the borders. The association of the blue (or yellow) border with reward was assumed to lead to an automatic response when positive images appeared within the border akin to that of humans reading words more quickly when printed in the same color ink. The chimpanzees showed slower responses to images of veterinarians within the borders if they had recently been anesthetized—a negative experience for chimpanzees. This pattern of responding appeared to validate the procedure for assessing emotional responses to pictorial stimuli.

Others [18] later presented other chimpanzees, gorillas, and Japanese macaques (*Macaca mulatta*) with a similar task using images of preferred food items and snakes. The results were somewhat inconclusive with lower accuracies for positive/food items but only apes displaying lower accuracy to negative/snake items when compared to baseline. The procedure was since extended to examine the effects of biologically relevant social (i.e., facial expressions of unfamiliar bonobos that differed in valence) and non-social (i.e., foods, predators) stimuli in bonobos [19] (*Pan, paniscus*). These researchers predicted that bonobos would respond more slowly to both positive and negative items compared to neutral baseline items. Somewhat counterintuitively, they found the slowest responses to positive social stimuli. However, they found slower response latencies to negative nonsocial stimuli. They also predicted, but failed to find, lower accuracy for valenced compared to neutral stimuli. In all three previous studies using the emotional Stroop task with nonhuman apes, researchers compared the responses to positive and negative stimuli to responses to neutral baseline items. In the current study, we omitted the neutral category and directly compared responses to positive and negative items, although we compared performance on these test trials to performance on control trials like those used in the proof-of-concept phase [17].

The previous studies [17,18,19] presented the apes with a test phase in which the same image appeared within both borders on a single trial. In the current study, we presented two phases like this with two separate sets of positive and negative items. However, we also implemented a novel phase in which positive items were paired with each of the negative items and positive and negative items were presented together on each trial; half the time with the positive item appearing in the blue border (congruent) and half the time with the positive item appearing in the yellow border (incongruent). We hypothesized that the gorillas would show slower responses when negative compared to positive items were placed within the colored borders that had previously been associated with reward. This hypothesis was based on the reasoning that stimuli evoking positive emotional responses would not produce a processing conflict when placed within colored borders previously associated with food rewards because both the stimulus and the border would evoke positive emotions. However, if a stimulus evoking a negative emotional response was placed within a border associated with reward, this would create an approach/avoid conflict and cause response slowing and reductions in accuracy (choosing the correctly colored border). Thus, in our procedure, the positive stimulus appearing within the reinforced border was considered congruent and served as the baseline against which to compare responses to negative items presented within the reinforced border.

## 2. Methods

### 2.1. Subjects

Three male Western lowland gorillas (*Gorilla gorilla gorilla*), Chipua (Chip), Pendeke (Pende) and Kongo-Mbeli (Kongo), participated in testing between June 2017 and January 2018. The gorillas were half-brothers between 17 and 19 years of age at the time of testing. They lived in a bachelor group along with two male drills (*Mandrillus leucophaeus*) with intermittent contact (together most of the time but separated overnight several times a week and for an hour in the mornings for feeding and other husbandry-related activities) at the Detroit Zoo in Royal Oak, MI.

Testing took place three mornings a week between 07:00 and 08:00 or between 08:00 and 09:00 in a restricted access indoor area with each gorilla tested in an individual holding area where they spent about an hour every morning while their primary habitats were being serviced. During this period, the gorillas were tested in other tasks including a speeded response test of affect [28], and a conditional discrimination task [Vonk, unpublished data]. They had previously participated in tests of quantity estimation [29], matching to sample [30], food preference tests [6], Likert scale training [7], and various cognitive bias tests [31,32] all presented on a touchscreen. The study was reviewed and approved by the Oakland University Institutional Animal Care and Use committee (IACUC # 12063). All testing was voluntary and food rewards were approved by the veterinary staff at the Detroit Zoo.

### 2.2. Materials

The touchscreen tests were programmed in Inquisit v. 2.0 by Millisecond and presented on a Panasonic Toughbook Laptop CF19 projected to a 19” VarTech Armorall capacitive touchscreen monitor. The touchscreen monitor was welded onto a rolling LCD panel cart and covered by a wooden ceiling and sides. A 1.2 m by 1.2 m plywood ramp was placed in front of each gorilla’s indoor holding area so that the screen was flush with the steel mesh, through which the gorillas could touch the screen with one finger. The laptop was placed on the shelf behind the touchscreen, which was covered by the wooden box but accessible to the researcher (JV or MM) who stood behind the cart so that her face was not visible to the subject during the task.

Stimuli consisted of two-dimensional images drawn in Microsoft Paint and photographs taken by the researcher (JV) of objects familiar to the gorillas. All images were 500 × 360 pixels). The borders were drawn in a thickness of 5 px. Foods came from the gorillas’ regular morning meal (i.e., various fruits, vegetables, and primate biscuits). The gorillas were fed what remained of the food in the trays immediately after testing regardless of their level of participation. A PVC chute attached to both the left and right sides of the cart allowed the experimenter to deliver the food reward to the gorilla without any direct contact. 

### 2.3. Procedure

#### 2.3.1. Phase One

All phases consisted of a two alternative forced choice discrimination in which each trial presented a choice between touching a rectangle with a blue border or a rectangle with a yellow border arranged side by side to fill the screen (side counterbalanced). In Phase One, the inside of the rectangles was white, like the background of the touchscreen, such that they appeared as open borders. The gorillas could select only a single image on each trial. They were rewarded for selecting the blue border with an auditory tone that had previously been associated with reward and a small food reward placed by the researcher into the PVC chute on the side of the cart. The next trial appeared without delay. If they selected the yellow border, they heard a different auditory tone that had previously been associated with non-reward and the next trial commenced with no time-out or delay. The gorillas participated in approximately four 24-trial sessions a day until they reached a criterion of 80% accuracy for four consecutive sessions or 90% accuracy for two consecutive sessions.

#### 2.3.2. Phase Two

**Figure 1:**
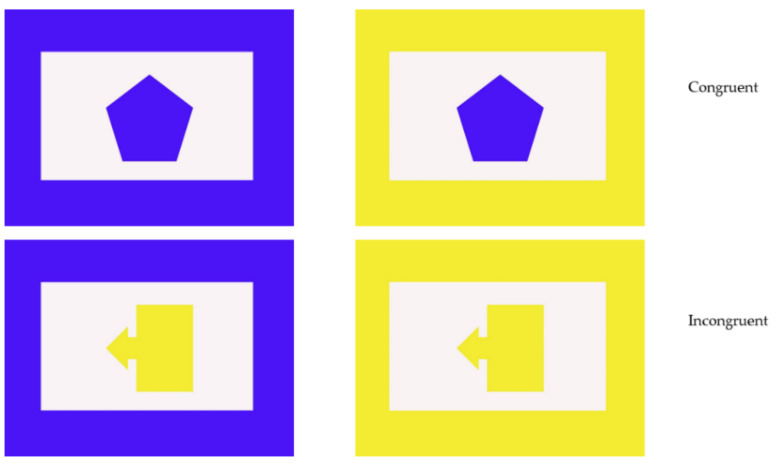
Example of Phase Two and control trials. Top row shows a congruent trial and bottom row shows an example incongruent trial. The blue border was always correct.

Phase Two consisted of 20 (Chip) or 30 sessions (Kongo, Pende) of 48 trials that were identical to trials in Phase One except that one of 24 two-dimensional shapes was placed inside each of the borders. Pende and Kongo were given an additional ten sessions because they were not choosing the blue rectangle on at least 70% of trials during the first 20 sessions. They reached this level of performance with an additional ten sessions. Twelve unique shapes were colored blue or colored yellow to match the rectangular borders to make up the 24 shapes (Figure 1). Each shape was presented inside both borders on two trials (counterbalanced for side) in random order within a session. The gorillas continued to be rewarded only for selecting the blue border, regardless of the color of the object inside. However, they were expected to respond more accurately and more quickly on congruent trials in which the shapes were blue. On incongruent trials, the shapes were yellow.

#### 2.3.3. Phase Three

**Figure 2:**
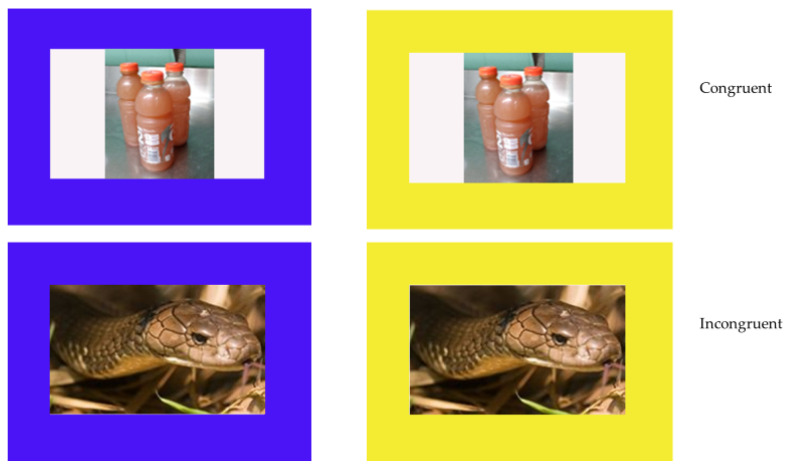
Example of Phase Three trials. Top row shows a congruent trial and bottom row shows an example incongruent trial. The blue border was always correct.

In Phase Three, we presented images that we selected to represent items that the gorillas were assumed to like (*n* = 6) and dislike (*n* = 6) within the blue and yellow borders along with control trials similar to those presented in Phase Two. The stimuli are available at the following link: OSF.IO/QV3BY. The gorillas completed 15 sessions, which consisted of 48 trials: 24 trials where each of six positive and six negative images were presented within both borders on a single trial and 24 control trials identical to those in Phase Two. On the control trials, each shape was presented once with the blue and yellow shapes appearing on the left on half of the trials and on the right for the rest of the trials. On the test trials, each image was presented within both borders on two trials with side counterbalanced (Figure 2). Gorillas were expected to respond more accurately and more quickly on congruent trials in which the positive items were presented within both borders compared to incongruent trials in which the negative items were presented in both borders. Trials were presented in random order.

#### 2.3.4. Phase Four

**Figure 3:**
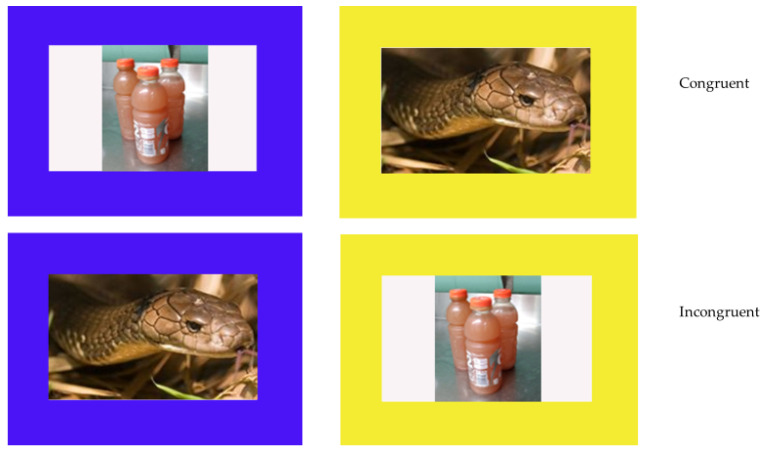
Example of Phase Four trials. Top row shows a congruent trial and bottom row shows an example incongruent trial. The blue border was always correct.

Phase Four consisted of 60 trial sessions with 24 control trials (like those presented in Phase Three), and 36 trials in which each of the six positive images were paired with each of the six negative images used in Phase Three. Positive images were presented either in the blue (18 congruent trials) or yellow borders (18 incongruent trials), with the negative item in the opposite colored border (Figure 3). Each image was presented six times (three times in the blue border and three times in the yellow border) paired once with each of the six opposite valence items. Within each trial type (congruent, incongruent), the blue border appeared on the left and right an equal number of times.

#### 2.3.5. Phase Five

This phase was the same as Phase Three with a new set of six assumed positive and six assumed negative items. The gorillas completed ten sessions of this phase.

#### 2.3.6. Phase Six

The gorillas completed ten sessions of this phase. This phase was the same as Phase Four with the items used in Phase Five except that a photograph of the inside habitat was removed and replaced with a photograph of a squirrel. A photograph of an empty food tray had been considered a negative item in Phase Five, but after observing that this was an outlier in terms of the response times among the negative items, we reconsidered that even empty food trays were associated with food and might therefore be better conceived of as positive items. Therefore, the empty food tray was reclassified as a positive item in Phase Six. Similarly, a photograph of the outdoor habitat had been classified as a positive item in Phase Five, but based on response time analysis, it was reclassified as a negative item in Phase Six. These changes occurred after Pende had completed a single session of Phase Six, Kongo had completed two sessions and Chip had completed three sessions. Thus, these six sessions were not included in analyses.

### 2.4. Statistical Analyses

We removed data from trials where latencies to respond were greater than two standard deviations above the mean for that type of stimuli (e.g., congruent control trials, incongruent test trials) for each subject. We also removed any response latencies below 100 ms based on our assessment that such responses happened too quickly for the subject to have attended to the stimuli. All statistical analyses were conducted using SPSS v. 28. For each phase of the study, we used generalized linear mixed models (GLMM). We analyzed the latencies to respond with a loglinear distribution because the data were skewed right. We also used GLMMs to analyze accuracy using a binomial distribution with a loglink function because each trial was scored as correct or incorrect. In all analyses, we included subject as a random factor and trial type (control, test) and congruence (congruent, incongruent) and their interaction as fixed factors. Phase Two used only a single trial type (control trials) so type was not included in analyses for Phase Two.

## 3. Results

### 3.1. Phase One

The gorillas reached criterion in 11 (Kongo), 13 (Chip), and 14 (Pende) sessions (264–336 trials, 304 trials on average). We did not perform analyses on these data as this phase was simply to train gorillas to associate the blue border with food reward and to associate the yellow rectangle with the lack of reward.

### 3.2. Phase Two 

#### 3.2.1. Response Latencies

There was a significant effect of congruence on response time, but the gorillas responded more quickly on incongruent (*M* = 2043.243, *SEM* = 681.337) compared to congruent trials (*M* = 2226.291, *SEM* = 742.376), which was in the opposite direction from what was predicted (*F* _1, 3623_ = 14922.636, *p* < 0.001). However, when examining the latencies separately for each gorilla in GLMs with gamma log link distributions, Kongo responded more quickly on congruent than incongruent trials (*χ*^2^ = 4.483, *p* = 0.034). Chip (*χ*^2^ = 7.068, *p* = 0.008) and Pende (*χ*^2^ = 6.785, *p* = 0.009) both responded more quickly on incongruent trials, which was opposite to the predicted direction.

Although we analyzed data from all trials, the same pattern of results emerged if we analyzed only trials on which subjects were correct, with the gorillas overall still responding more quickly on incongruent trials (*M* = 2423.654 versus 2706.920, *F* _1, 2615_ = 7.169, *p* = 0.007), and there no longer being a significant effect of congruency on latencies to respond for Kongo (*χ*^2^ = 2.229, *p* = 0.135).

#### 3.2.2. Response Accuracy

There was a significant effect of congruence on accuracy. The gorillas were more accurate on congruent (*M* = 0.787, *SEM* = 0.098) compared to incongruent trials (*M* = 0.753, *SEM* = 0.108), which was as predicted (*F*_1, 3623_ = 6.149, *p* = 0.013).

### 3.3. Phase Three

#### 3.3.1. Response Latencies

**Figure 4:**
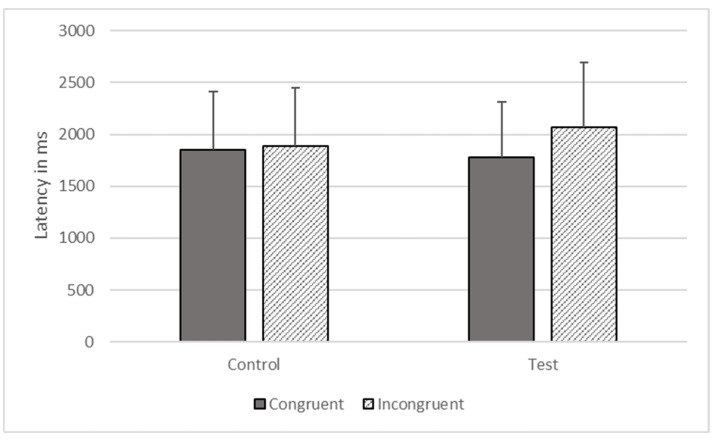
Response latencies as a function of trial types in Phase Three.

There were significant effects of type (*F*_1, 1806_ = 602.734, *p* < 0.001) and congruence (*F*_1, 1806_ = 6446.100, *p* < 0.001) on response time, but these effects were qualified by their interaction (*F*_1, 1806_ = 4156.345, *p* < 0.001). The gorillas responded more quickly on congruent (*M* = 1775.274, *SEM* = 535.562) compared to incongruent trials (*M* = 1973.892, *SEM* = 624.191) on test trials, but there was no significant difference between congruent (*M* = 1851.851, *SEM* = 558.664) and incongruent trials (*M* = 1883.101, *SEM* = 568.091) on control trials (see Figure 4).

#### 3.3.2. Response Accuracy

There was a significant effect of trial type (*F*_1, 1806_ = 4.293, *p* = 0.038) on accuracy. The gorillas responded more accurately on control (*M* = 0.816, *SEM* = 0.105) compared to test trials (*M* = 0.778, *SEM* = 0.120), but there were no effects of congruence and no interaction.

### 3.4. Phase Four

#### 3.4.1. Response Latencies

**Figure 5:**
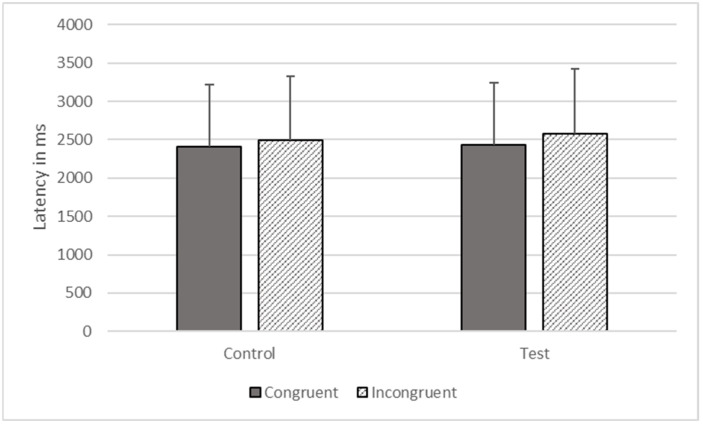
Response latencies as a function of trial types in Phase Four.

There were significant effects of type (*F*_1, 3414_ = 806.178, *p* < 0.001) and congruence (*F*_1, 3414_ = 4549.353, *p* < 0.001) on response time, but these effects were qualified by their interaction (*F*_1, 3414_ = 240.982, *p* < 0.001). The gorillas responded more quickly on congruent (*M* = 2432.752, *SEM* = 806.202) compared to incongruent test trials (*M* = 2572.064, *SEM* = 852.369), but there was no difference between congruent (*M* = 2411.843, *SEM* = 799.273) and incongruent control trials (*M* = 2497.391, *SEM* = 827.623, see Figure 5).

#### 3.4.2. Response Accuracy

**Figure 6:**
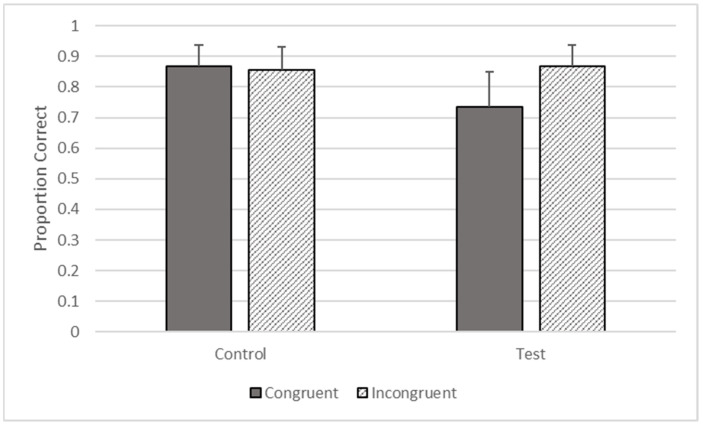
Proportion of trials correct as a function of trial types in Phase Four.

There were significant effects of type (*F*_1, 3414_ = 16.841, *p* < 0.001) and congruence (*F*_1, 3414_ = 15.312, *p* < 0.001) on accuracy, but these effects were qualified by their interaction (*F*_1, 3414_ = 25.797, *p* < 0.001). The gorillas responded more accurately on incongruent (*M* = 0.867, *SEM* = 0.069) compared to congruent trials on test trials (*M* = 0.735, *SEM* = 0.116), but there was no difference between incongruent (*M* = 0.856, *SEM* = 0.074) and congruent control trials (*M* = 0.869, *SEM* = 0.069, see Figure 6).

### 3.5. Phase Five

#### 3.5.1. Response Latencies

**Figure 7:**
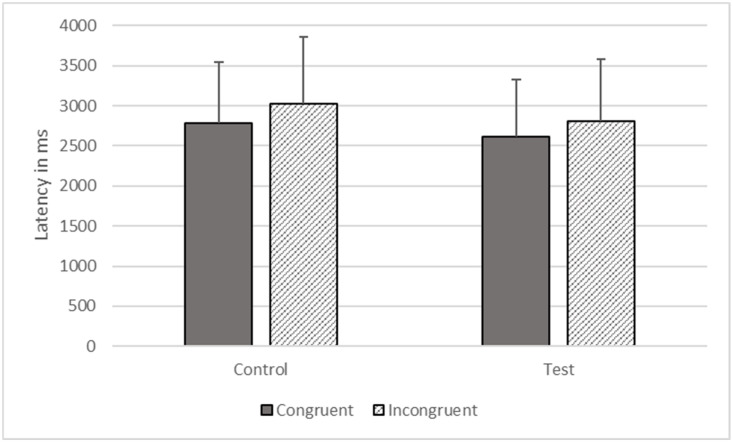
Response latencies as a function of trial types in Phase Five.

There were significant effects of type (*F*_1, 1368_ = 5074.203, *p* < 0.001) and congruence (*F*_1, 1368_ = 6509.927, *p* < 0.001) on response time, but these effects were qualified by their interaction (*F*_1, 1368_ = 17.723, *p* < 0.001). The gorillas responded more quickly on congruent (*M* = 2695.486, *SEM* = 738.053) compared to incongruent trials (*M* = 2917.413, *SEM* = 798.819), as predicted, but the difference was larger for control than for test trials (see Figure 7).

#### 3.5.2. Response Accuracy

There was a significant effect of trial type (*F*_1, 1368_ = 7.248, *p* = 0.007) on accuracy. The gorillas responded more accurately on control (*M* = 0.922, *SEM* = 0.075) compared to test trials (*M* = 0.883, *SEM* = 0.107), but there were no effects of congruence and no interaction.

### 3.6. Phase Six

#### 3.6.1. Response Latencies

**Figure 8:**
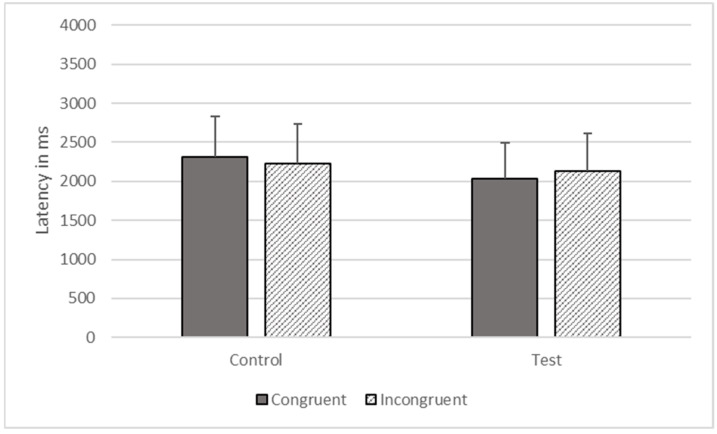
Response latencies as a function of trial types in Phase Six.

There were significant effects of type (*F*_1, 1473_ = 5991.633, *p* < 0.001) and congruence (*F*_1, 1473_ = 17.342, *p* < 0.001) on response time, but these effects were qualified by their interaction (*F*_1, 1473_ = 1491.423, *p* < 0.001). The gorillas responded more quickly on congruent (*M* = 2027.495, *SEM* = 464.530) compared to incongruent trials (*M* = 2126.589, *SEM* = 487.233), on test trials, but they responded more quickly to incongruent (*M* = 2220.597, *SEM* = 508.774) than congruent trials (*M* = 2307.586, *SEM* = 528.705) on control trials (see Figure 8).

#### 3.6.2. Response Accuracy

There was a significant effect of trial type (*F*_1, 1473_ = 19.684, *p*. < 0.001) on accuracy. The gorillas responded more accurately on control (*M* = 0.943, *SEM* = 0.049) compared to test trials (*M* = 0.883, *SEM* = 0.093), but there were no effects of congruence and no interaction.

## 4. Discussion

The application of tasks designed to assess the effects of emotional processing on human cognitive processes to study nonhuman emotion is a relatively new development in animal welfare science [33,34]. Researchers have adapted tests of cognitive bias [25,28,31,32,35], the dot probe task [36,37,38,39,40,41,42], speeded response procedures [25,43,44,45], and most recently, the modified emotional Stroop task [17,18,19] used here. These tasks still require validation due to the recency of their use, sometimes unexpected patterns of results, and alternative interpretations of response patterns [39,46]. Our results provide some validation of the emotional Stroop task to assess the emotional salience of pictorial stimuli for nonhuman apes, but do not align perfectly with findings from other applications of the task. Unlike previous findings [18,19], the gorillas made more errors in the incongruent condition of the validation phase, as predicted, but like bonobos tested previously [19], as a group, they did not respond more slowly in the incongruent compared to the congruent condition. Although this phase was originally designed as a proof of concept [17], and we did not obtain the expected results for latencies overall, we proceeded with the other planned phases of our experiment based on the accuracy results and the previous suggestion that Stroop effects on reaction times may be difficult to measure [47]. Others [19] also proceeded to test their subjects with test stimuli despite not obtaining the predicted results on these control trials.

As other researchers have pointed out [19], the congruent/incongruent distinction on these control trials may be complicated by the fact that the incongruent trials in the original study [17] presented greater contrast between the embedded object and the border on incongruent than congruent trials, but this was not confounded across the correct and incorrect choices. In contrast, in our design, as in the other two relevant studies, [18,19], the same blue or yellow stimulus was placed within both borders on each trial so that the incorrect choice (e.g., blue object in a yellow border) had greater contrast than the correct choice on congruent trials but the correct choice (yellow object in a blue border) had greater contrast on incongruent trials. However, one might expect that our method for the control trials would actually be more likely to lead to slower responding on incongruent than congruent trials for this reason. We used the design of [18,19] because we wanted to hold the embedded image constant and vary only the borders to reinforce the subjects’ focus on the border color as the cue to which stimulus was correct to further automatize this response. This procedure of placing the same image in both borders on a given trial was also consistent with the test trials in all three of the previous studies and in Phases Three and Five here, which allowed for comparison between control and test trials. Others analyzed latencies for only correct trials [17,19]. We analyzed data from all trials, but the pattern of results was the same either way. We chose to include latencies for incorrect trials because reductions in accuracy should contribute to the general slowing of response that we anticipated for negatively valenced items relative to positively valenced items. We did find the expected pattern of results for the emotionally valenced items, which might validate that they are more salient and therefore distracting than the control stimuli.

We presented our gorillas with up to 720 trials in Phase Two, whereas the original study [17] presented chimpanzees with only 80 test trials like the ones presented in Phase Two here, along with 40 control trials, not used here. It is possible that there were some habituation effects in our study that led to a reduction in latency effects over trials. When we examine only the first 10 sessions (240 trials), we still do not obtain the predicted effect of longer latencies for incongruent trials across all three subjects. Chimpanzees responded more slowly to negative stimuli only when only the first session was considered [17], but we found slower latencies to respond to negative stimuli across all phases when all sessions were included in the analyses. However, it is possible that there was habituation to the control stimuli across phases as we used the same control trials in each phase.

Other researchers [18] also pointed out that familiarity with touchscreen tasks may lead to ceiling effects for response time data. Although our subjects were also quite experienced with touchscreen tasks, having been tested three times a week for several years at the time of this study, we think this is an unlikely explanation for our results as we did find effects of response latencies using a different speeded response task [45]. Although Phase Two was designed to show that embedded images affected selection of the target borders as in the original study [17], we speculate that the borders were so salient and the task was so easy for our gorillas compared to other tests we had presented to them, e.g., [7,28,30,31], that they were not distracted by arbitrary two-dimensional shapes in blue and yellow that held no ecological validity. When we presented meaningful images of stimuli that we assumed had positive or negative valence for them, we did observe the expected response slowing when negative items were embedded within the positively reinforced blue border. That is, we did not observe habituation to our test stimuli.

There has been much debate in the literature regarding emotional Stroop effects in humans, as to which cognitive processes are affected [22,27,48,49,50,51,52,53,54], leaving interpretation of the results somewhat ambiguous. In some ways, it is not entirely surprising that effects in various studies using the pictorial emotional Stroop task with nonhuman primates are equivocal, because, even in humans, the pictorial version of the task leads to mixed patterns of results, e.g., [55,56,57]. Of course, it is not possible to use the verbal version of the task in nonverbal organisms, so it remains worthwhile to assess the validity of the nonverbal version of the task. It should be noted that the pictorial version of the emotional Stroop task modified for use with nonhumans [17] differs from the original emotional Stroop task used with humans in important ways. For example, in the original version of the task, the participant reads a single word and comparison of response times depending on whether the stimulus is neutral or emotionally salient is the critical measure. In the nonhuman primate version of the task, the participant is choosing between a motor response to two stimuli presented simultaneously so response times may also be impacted by the contrast between the two stimuli as much as between elements within a single stimulus.

Slight variations in procedures may lead to different patterns of response, especially for relatively small effects. The design of our study differed from previous versions of the emotional Stroop task presented to nonhuman primates. Our training phase involved only the blue and yellow borders and did not involve any other images. We did not present the gorillas with a transfer test because of this. We also presented a total of 12 positive and 12 negative stimuli representing different categories of objects (e.g., foods, habitats, other animals, keepers, objects) across four phases of the experiment, whereas previous studies [17,18,19] used only a single positive or negative category (e.g., snakes versus foods). Although our classification of items as positive or negative was subjective, we used analysis of response times to assess whether individual items behaved in a manner consistent to the general category (positive, negative). After adjusting for some possible misinterpretations after Phase Five, the response times for the group of items as a whole were consistent with our subjective classifications.

Most notably, others [17,18,19] presented neutral or control stimuli in addition to the negatively and positively valenced stimuli. For example, they used a blank white square within the border as a control stimulus in testing, whereas we used similar stimuli in training only. These researchers compared latencies and accuracy for both positive and negative stimuli to the neutral and baseline stimuli rather than directly comparing responses between positive and negative stimuli. In [19], bonobos exhibited longer response latencies on both positive and negative social stimuli in comparison to neutral stimuli. Interestingly, they responded the most slowly with positively valenced social stimuli. This is consistent with a growing body of work showing the processing effects of positive, as well as negative, emotionally salient stimuli. However, responses to the positive stimuli appear to depend upon subjects’ current motivational states [58]. Rather than comparing processing speed for positive and negative stimuli to neutral stimuli, we compared latencies between positive and negative stimuli directly and found that, in all phases, gorillas responded more slowly when negative stimuli were presented within borders associated with reward compared to when positive stimuli were presented within the same borders. We believe this finding validates our categorization of test stimuli as positively and negatively valenced. Our different analytical approach was because we were more concerned with developing a procedure for evaluating gorillas’ responses to particular familiar objects rather than attempting to assess overall emotional affect. Furthermore, we believe that placing a negative versus a positive image within borders associated with food or not creates an analog to the original emotional Stroop where either neutral or negative words appear in neutral colors. Our method presented a logical contrast where the positive image within the reinforced border served as the baseline against which to compare the effects of negative stimuli.

Expected effects of response slowing to negatively valenced items occurred regardless of whether the same object was presented in both borders on each trial (as in Phases Three and Five) or when a positive object was paired with a negative object on each trial (as in Phases Four and Six). These latter phases are a unique feature of our study. Others have not included a condition in which a positively valenced item was presented alongside a negatively valenced item. We believe this novel condition sets up a conflict analogous to the process captured by the original Stroop test where the subject must choose between a preferred and non-preferred item to touch, which is sometimes in conflict with the trained response (incongruent trials) and sometimes not (congruent trials). In the more commonly presented test trials (our Phases Three and Five), there is only one image to touch because the same image appears in both borders, making it easier to ignore the identity of the object and focus on the border colors as they are the only element that differs between the two options.

Our results are more consistent with bonobos’ responses to nonsocial rather than social stimuli [19], which makes sense given that we also used nonsocial stimuli for the most part. However, it is unclear why social and nonsocial stimuli should lead to different patterns of responding in the emotional Stroop task. Previously, we used social stimuli similar to that used by [19] in a speeded response task where the same gorillas simply had to touch an image of an unfamiliar [45] or familiar gorilla [Vonk & Truax, in preparation] staring straight ahead or with gaze averted. In these studies, the gorillas showed inconsistent patterns of response to threatening stimuli, sometimes showing the predicted response slowing effects and sometimes not. It is possible that social stimuli are not optimal for two-dimensional presentation in the absence of other salient cues such as movement, olfactory and auditory cues. Whereas non-social stimuli are meaningful by virtue of their identity alone, for social stimuli, the identity of the individual may not be inherently positive or negative in the absence of contextual cues as to the individual’s mood or behavior, which cannot be captured using two-dimensional stimuli.

It should be noted that the gorillas acquired the basic color discrimination task in far fewer trials than the chimpanzees tested previously [17]. The gorillas tested here acquired the discrimination in an average of 304 trials, whereas the gorillas tested previously took an average of 1853 trials [18]. Chimpanzees took over 2000 trials on average [18] and four other chimpanzees never reached the criterion in 4000 trials [17]. However, it is also important to note that the training used here did not involve potentially distracting images within the borders. The colors blue and yellow are likely highly salient to the gorillas when comparing their performance on this task to other tasks we had presented to them previously, e.g., [28,30,31]. It is also possible that the high level of performance was an artifact of correctly rewarding them for selecting the darker color that stood out in stronger contrast against the white background of the touchscreen.

The lack of effects of congruence on accuracy may have resulted because of the high levels of performance of the gorillas in our task. That gorillas were close to ceiling on the control trials may have led to the few effects of accuracy in the later test phases. That accuracy was higher on control trials, compared to test trials, confirms our prediction that the emotionally valenced, but not neutral, stimuli interfered with performance. Thus, we think the procedure was useful for corroborating our impressions of gorilla preferences.

However, our study had several limitations. First, we had a very small sample of gorillas, although the number of subjects was comparable to the previous studies investigating emotional Stroop processes in nonhuman primates [17,18,19]. Second, we presented a fairly large number of trials across all phases of the experiments, which is likely to have led to some habituation to stimuli. Others [59,60] have suggested that humans may habituate in the emotional Stroop task after repeated presentation of the same stimuli and have recommended presenting the stimuli in blocks [50]. The recommendation to present blocks of positive and negative stimuli is also made based on findings that response slowing may be due to the stimulus presented in the previous, rather than the current trial [27,50]. We did not block our stimuli; however, we used a larger number of unique stimuli and each stimulus was presented no more than six times in a single session. In addition, at least 40% of the trials in our test sessions consisted of control trials, which reduces carry-over [61] and habituation. Future studies should examine the nature of the preceding stimulus, however, and potentially plan to analyze responses to neutral stimuli following positive and negative stimuli and positive and negative stimuli following neutral stimuli [50]. It is somewhat surprising that we observed results consistent with response slowing to negative relative to positive stimuli given the design of our study when previous studies using a blocked design [18,19] did not find the expected patterns of results, but it is possible that this is because the high proportion of control trials interspersed in our test sessions reduced carry-over.

## 5. Conclusions

Our study is the fourth to use the emotional Stroop task with nonhuman primates, and the second to do so with gorillas. It adds to the ambiguity in interpreting results from such tasks given the lack of consistent findings with accuracy and latency to respond to congruent and incongruent trials. We would caution researchers to use this task only when stimuli with very explicit (and externally validated) emotional valances can be identified. It is probably not a task suitable for exploring emotional responses to stimuli for which emotional responses are unknown. Although, it would be possible to assess response time patterns for individual stimuli to potentially identify whether stimuli led to effects consistent with having negative or positive valences. The task is easier for subjects to learn compared to other methods, such as judgement bias tasks, and may therefore be a useful tool with which to identify individual differences in susceptibility to threatening stimuli [54]. Most significantly, authors have recently argued [62] that animal species responding to valenced stimuli allows for the attribution of emotions to these species—a notable advancement in the study of animal emotion. We believe we have shown evidence for meaningful responses to valenced stimuli here, thereby validating the assumption that gorillas are emotional beings.

## Data Availability

The data and images presented in this study are available here: OSF.IO/QV3BY.
